# Author Correction: Genus-wide sequencing support a two-locus model for sex-determination in *Phoenix*

**DOI:** 10.1038/s41467-018-07742-5

**Published:** 2018-12-03

**Authors:** Maria F. Torres, Lisa S. Mathew, Ikhlak Ahmed, Iman K. Al-Azwani, Robert Krueger, Diego Rivera-Nuñez, Yasmin A. Mohamoud, Andrew G. Clark, Karsten Suhre, Joel A. Malek

**Affiliations:** 1 Department of Genetic Medicine, Weill Cornell Medicine-Qatar, PO Box 24144, Doha, Qatar; 2 Department of Biological Sciences, University of Cincinnati, 45221 Cincinnati, OH USA; 3 Genomics Laboratory, Weill Cornell Medicine-Qatar, PO Box 24144, Doha, Qatar; 4 Department of Physiology and Biophysics, Weill Cornell Medicine-Qatar, PO Box 24144, Doha, Qatar; 5 USDA-ARS National Clonal Germplasm Repository for Citrus & Dates, 92507 Riverside, CA USA; 6 Department of Plant Biology, Faculty of Biology, University of Murcia, 30100 Murcia, Spain; 7 Department of Molecular Biology and Genetics, Cornell University, 14853 Ithaca, NY USA

Correction to: *Nature Communications*; 10.1038/s41467-018-06375-y; published online 28 Sept 2018

In the original version of this Article, the affiliation of the first author, Maria F. Torres, ‘Department of Biological Sciences, University of Cincinnati, Cincinnati, 45221, OH, USA’ was incorrectly assigned as a present address and should have been listed as a full affiliation. This error has been corrected in both the PDF and HTML versions of the Article.

