# Supplementary material for: Genus-wide sequencing supports a two-locus model for sex-determination in Phoenix
Source: Nat Commun. 2018 Sep 28;9:3969. doi: 10.1038/s41467-018-06375-y (PMC6162277; doi:10.1038/s41467-018-06375-y)
Supplement: Supplementary file 1 — Supplementary Information [file 41467_2018_6375_MOESM1_ESM.pdf]

Genus-wide sequencing supports a two-locus model for sex-determination in *Phoenix*

Torres *et al.*

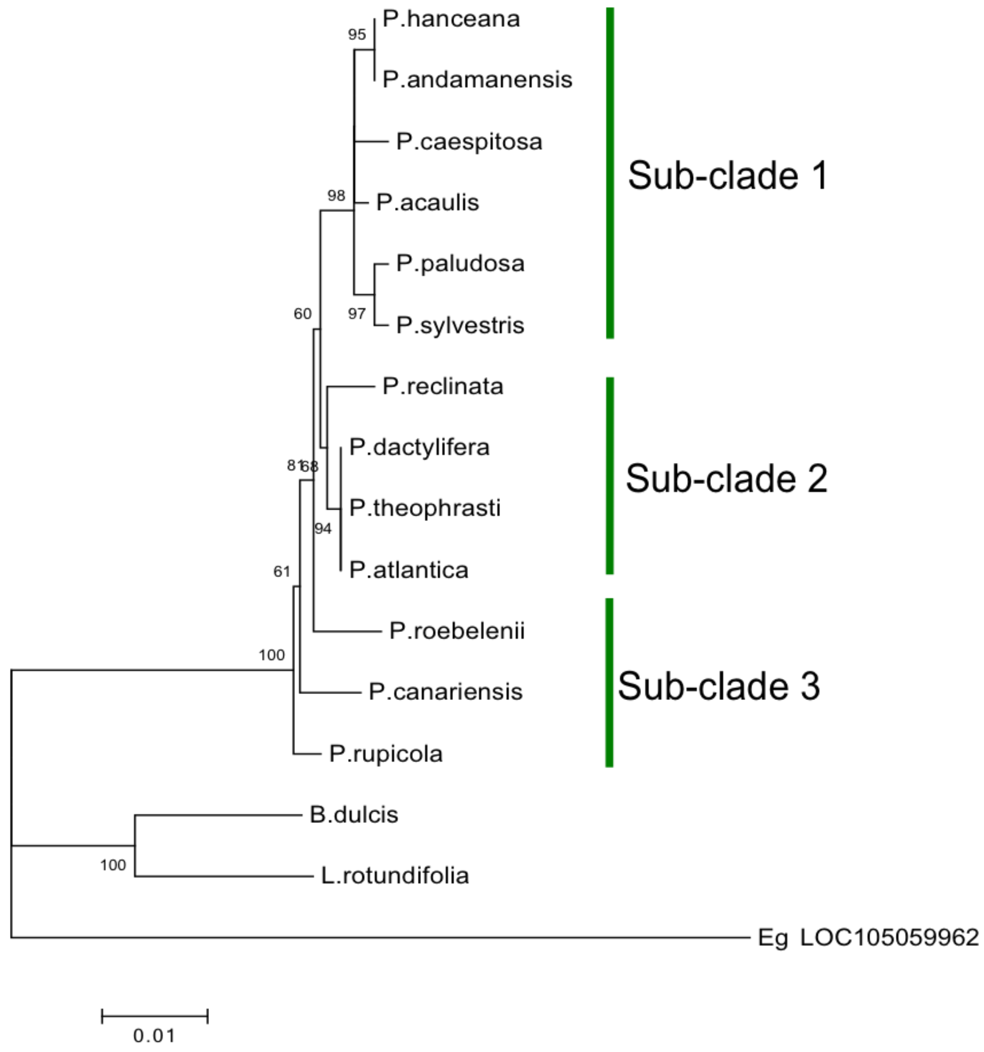

Supplementary Figure 1. Cyp703 phylogeny. Maximum likelihood phylogenetic tree based on alignment of k-mer derived *CYP703* cDNA sequences from *Phoenix* and the hermaphrodites *Brahea* and *Livistona*. Branch support values at the nodes (if >50%) were obtained by 1000 bootstrap replications. Branch lengths represent number of substitutions per site. The oil palm *CYP703A* sequence (Eg_LOC105059962) was utilized to infer *de novo* sequences from other palm species. Eg= *Elaeis guineensis*. *B. dulcis*= *Brahea dulcis*. *L. rotundifolia* = *Livistona rotundifolia*

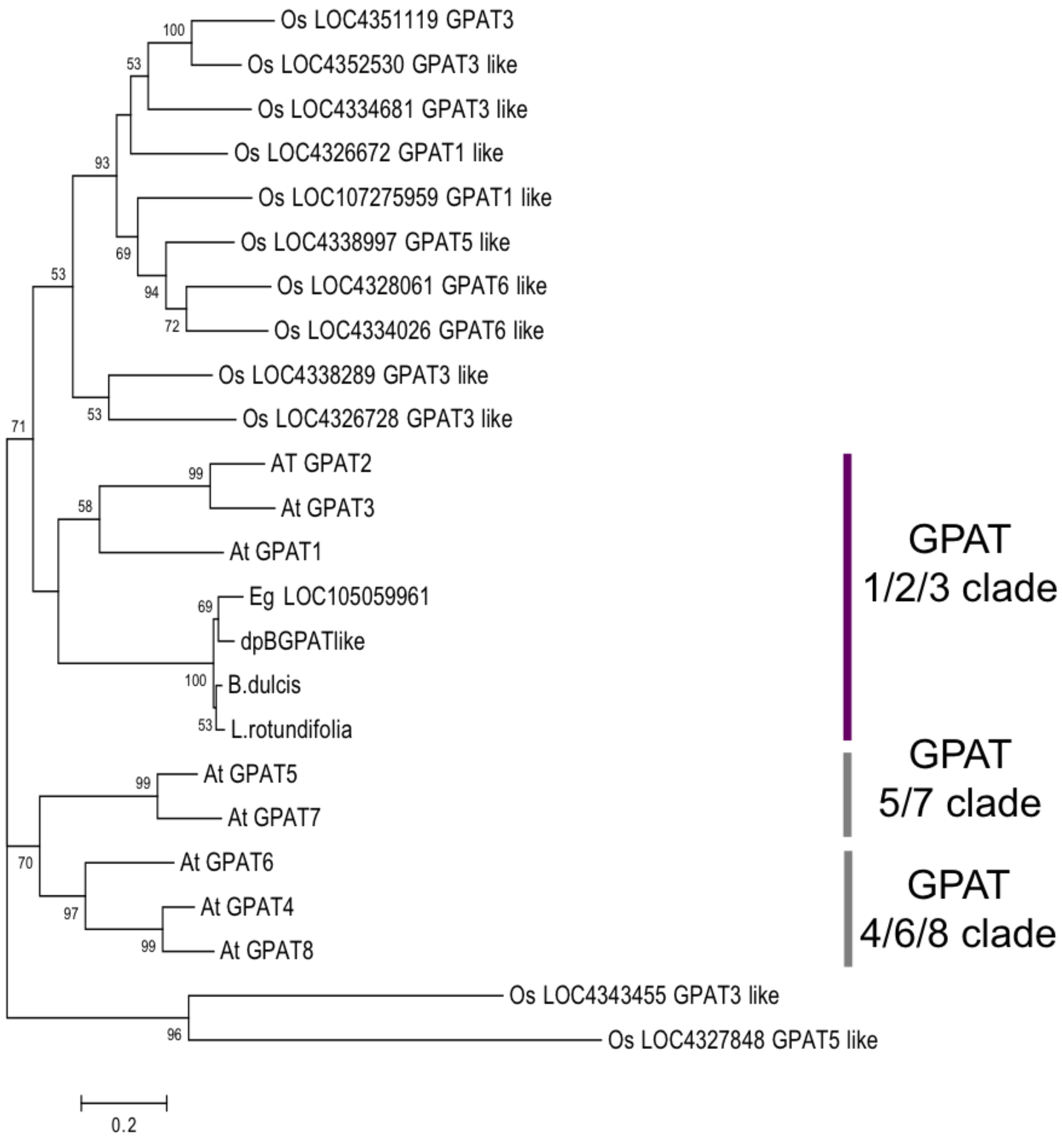

Supplementary Figure 2. GPAT phylogeny. Maximum likelihood phylogenetic tree based on alignment of GPAT sequences from Arabidopsis, rice and k-mer inferred sequences from *P. dactylifera*, *Brahea* and *Livistona*.

Branch support values indicated at the nodes (if >50%) were obtained by 1000 bootstrap replications. Branch lengths represent number of substitutions per site. The oil palm *GPAT* sequence (Eg_LOC105059961) was utilized to infer *de novo* GPAT sequences from other palm species. Arabidopsis and rice sequences were described by Beisson *et al.* [1] and Men *et al.* [2]. Os= *Oryza sativa*. At=*Arabidopsis thaliana*.. Eg= *Elaeis guineensis*. *B. dulcis*= *Brahea dulcis*. *L. rotundifolia* = *Livistona rotundifolia*. dpBGPATlike= date palm kmer *GPAT* gene

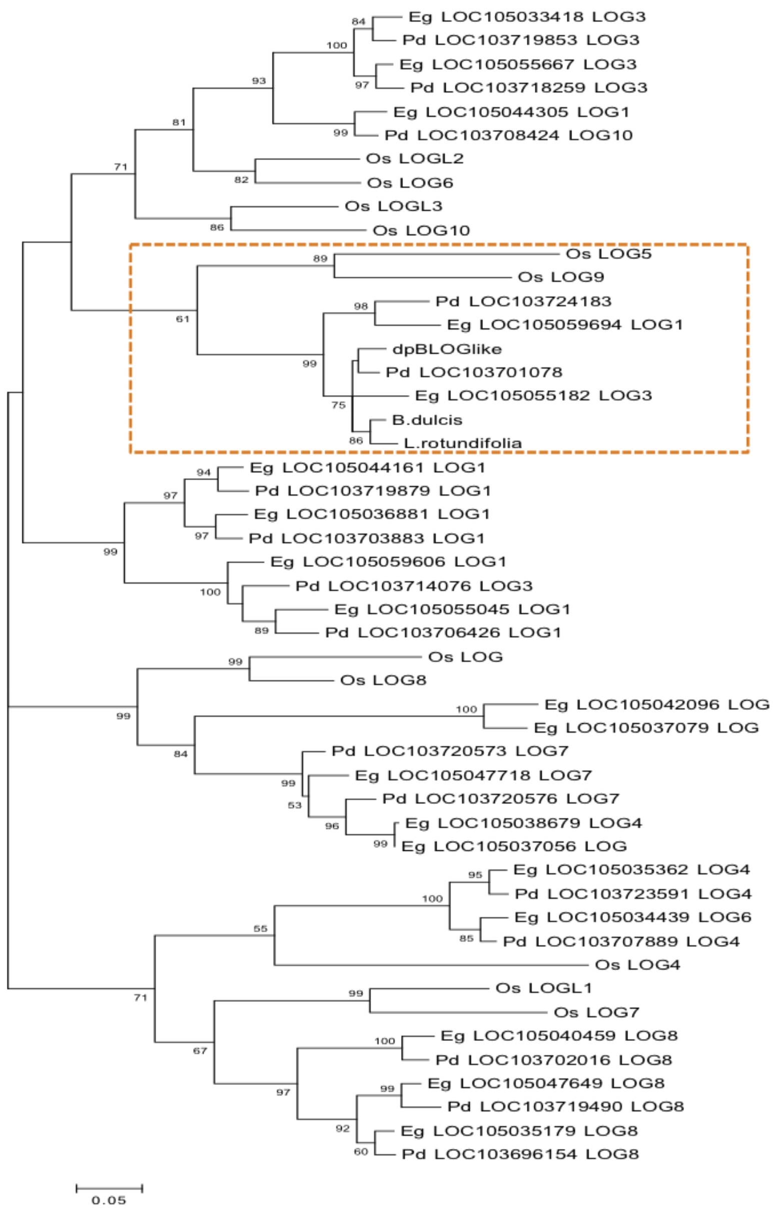

Supplementary Figure 3. LOG phylogeny. Maximum likelihood phylogenetic tree based on alignment of all LOG sequences from oil palm, date palm, rice and k-mer inferred sequences from *P. dactylifera, Brahea and Livistona*. Branch support values indicated at the nodes (if >50%) were obtained by 1000 bootstrap replications. Branch lengths represent number of substitutions per site. The oil palm LOG sequence (Eg_LOC105055182) was utilized to search the NCBI database for similarities in the entire date palm and oil palm genomes. Os= Oryza sativa. Pd= *Phoenix dactylifera*. Eg= *Elaeis guineensi*. *B. dulcis= Brahea dulcis. L. rotundifolia = Livistona rotundifolia*. dpBLOGlike= date palm male-specific kmer *LOG* gene.

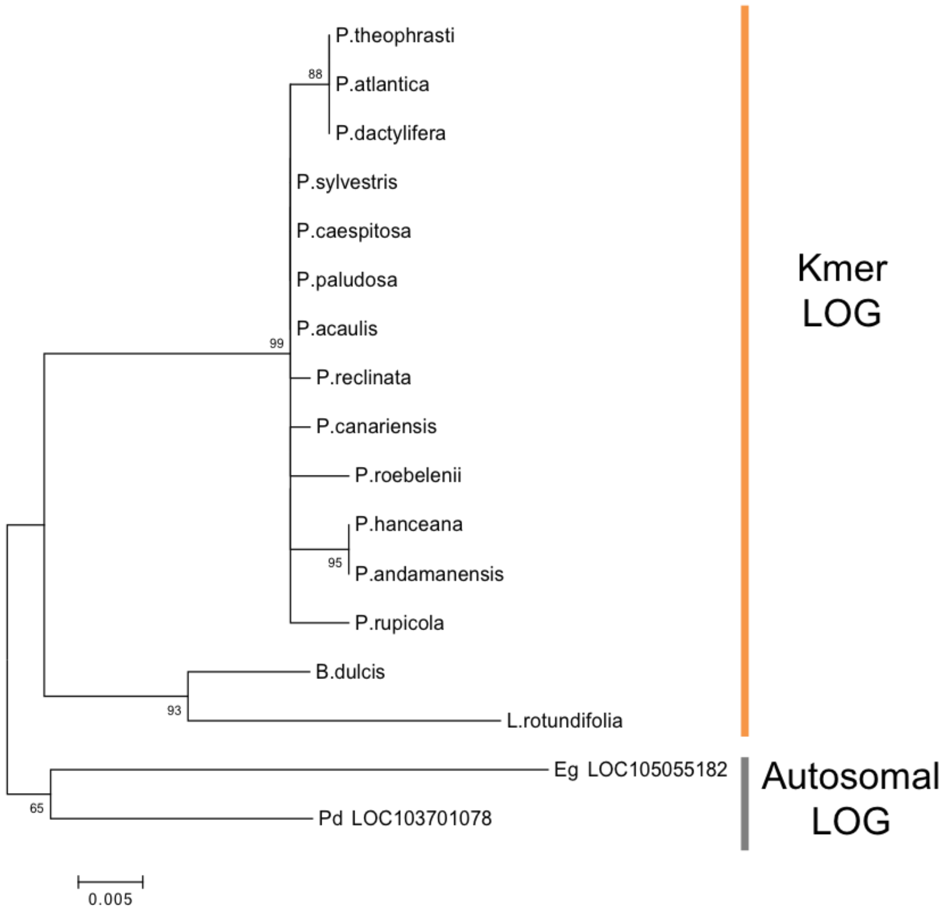

Supplementary Figure 4. Autosomal and male-specific LOG phylogeny. Maximum likelihood phylogenetic tree based on alignment of k-mer derived LOG sequences from *Phoenix* and the hermaphrodites *Brahea* and *Livistona*. Branch support values indicated at the nodes were obtained by 1000 bootstrap replications. Branch lengths represent number of substitutions per site The Oil Palm LOG sequence (Eg_LOC105055182) was utilized to infer *de novo* sequences from other palm species. The *P. dactylifera* autosomal LOG (Pd_LOC103701078) was included for reference. Eg= *Elaeis guineensis*. *B. dulcis*= *Brahea dulcis*. *L. rotundifolia* = *Livistona rotundifolia*

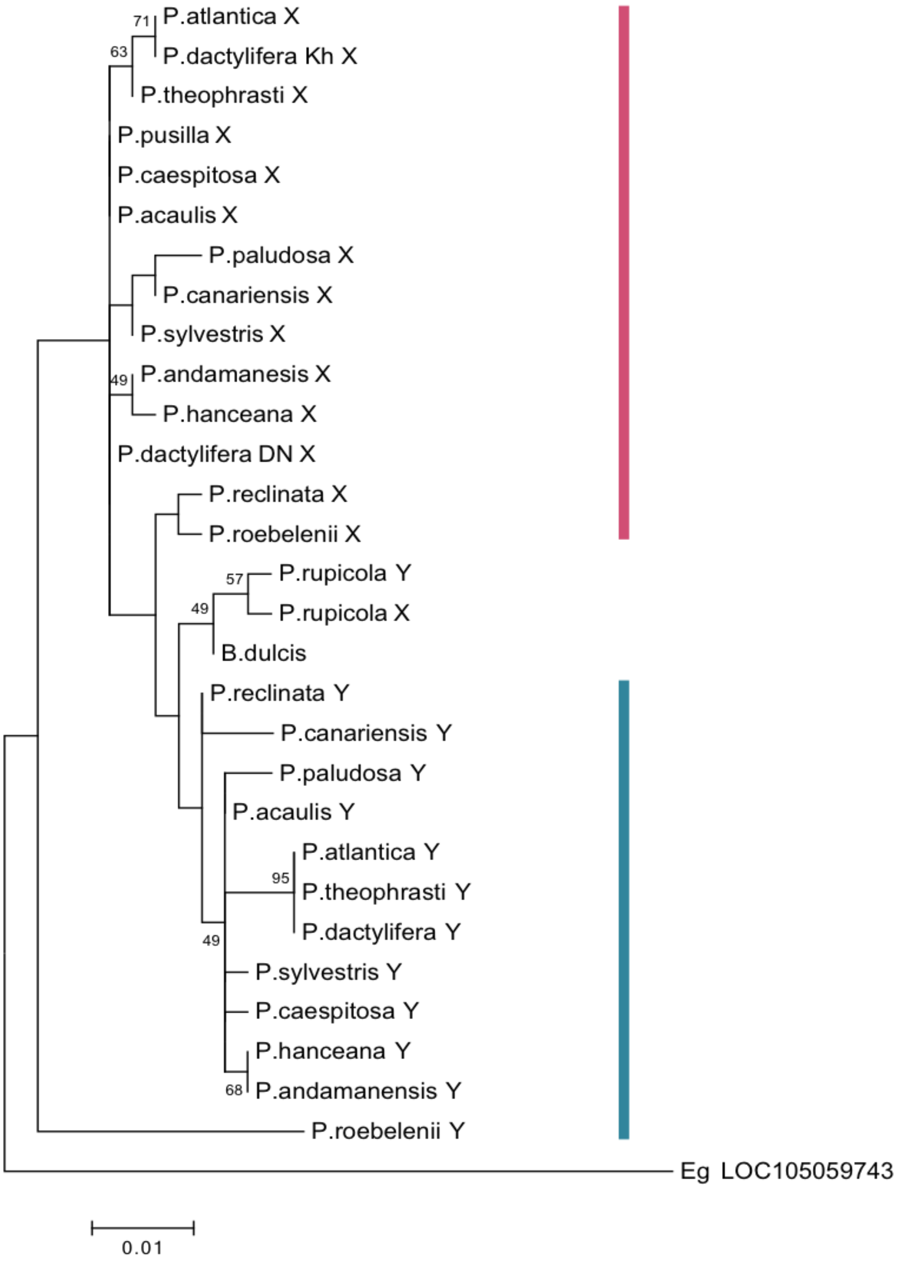

Supplementary Figure 5. Cytidine deaminase phylogeny. Maximum likelihood phylogenetic tree based on alignment of kmer derived cytidine deaminase cDNA sequences from *Phoenix* and Brahea. Branch support values at the nodes (if >40%) were obtained by 1000 bootstrap replications.

Branch lengths represent number of substitutions per site. Cytidine deaminase sequences were inferred *de novo* from sequenced male and female BACs. The oil palm cytidine deaminase gene (Eg_LOC105059743) was included as an outgroup. Eg= *Elaeis guineensis*. *B. dulcis*= *Brahea dulcis*. *P. dactylifera DN*= *P. dactylifera Deglet Noor*. *P. dactylifera Kh*= *P. dactylifera Khalas*. Male alleles for the genus clustered together in general (blue line – Y alleles, red line – X alleles)

Supplementary Table 1. A table with all samples used for kmer analysis and validation test in this study

Species	Sex	ID	Collection	Short name	Sequence coverage
**Used in kmer analysis**					
P. dactylifera (Deglet Noor)[*]	F	RIV 2154	USDA, CA	dnPdF	71.3517
P. dactylifera (Deglet Noor)	M	RIV 2165 PL	USDA, CA	dnPdM	67.531
P. dactylifera (Khalas)	F	khlsF	Qatar	khlsF2016	69.4486
P. roebilinii	F	RIV 2922 PL	USDA, CA	P03roeF	23.3215
P. roebilinii	M	RIV 2963 PL	USDA, CA	P5roeM	37.2331
P. canariensis	F	RIV 6839 PL	USDA, CA	P08canF	34.7572
P. canariensis	M	RIV 6840 PL	USDA, CA	P09canM	67.0642
P. hanceana (loureiroi?)	M	RIV 6823 PL	USDA, CA	P13hanM	28.1128
P. hanceana (loureiroi?)	F	RIV 6791 PL	USDA, CA	P14hanF	27.5131
P. paludosa	F	RIV 6817 PL	USDA, CA	P17palF	38.5184
P. paludosa	M	RIV 6819 PL	USDA, CA	P19palM	45.7222
P. reclinata	M	RIV 6807 PL	USDA, CA	P20recM	58.0921
P. reclinata	F	RIV 6811 PL	USDA, CA	P21recF	41.3914
P. sylvestris	F	RIV 2248 PL	USDA, CA	P23sylF	32.3765
P. sylvestris	M	RIV 7395 PL	USDA, CA	P25sylM	27.5639
P. andamanensis	F	N/A	UMH, Spain	Q01andF	36.6741
P. andamanensis	M	N/A	UMH, Spain	Q03andM	41.7865
P. caespitosa	F	N/A	UMH, Spain	Q07caeF	55.0238
P. caespitosa	M	N/A	UMH, Spain	Q09caeM	45.7855
P. acaulis	F	N/A	UMH, Spain	P02acaF	31.0049
P. acaulis	M	N/A	UMH, Spain	Q10acaM	29.0575
P. theophrastii	F	N/A	UMH, Spain	Q17theF	33.7925
P. theophrastii	M	N/A	UMH, Spain	Q19theM	93.2802
P. atlantica	F	N/A	UMH, Spain	Q22atlF	48.0664
P. atlantica	M	N/A	UMH, Spain	Q23atlM	48.9665
P. rupicola	F	N/A	UMH, Spain	P0XrupF	22.906
P. rupicola	M	N/A	UMH, Spain	Q15rupM	36.4488
P. pusilla	F	N/A	UMH, Spain	Q14pusF	30.7199
Brahea dulcis	Herm	N/A	Hunington Gardens, CA	braheaSpp	44.8707
Livistona *rotundifolia*	Herm	N/A	Hunington Gardens, CA	livistonaSpp	32.4906
**Used in validation test**					
P. acaulis	F	RIV 3363 PL	USDA, CA	P01acaF	38.33
P. roebilinii	F	RIV 2939 PL	USDA, CA	P04roeF	20.74
P. canariensis	F	RIV 6836 PL	USDA, CA	P06canF	31.86
P. canariensis	M	RIV 6837 PL	USDA, CA	P07canM	26.79
P. reclinata	F	RIV 2975 PL	USDA, CA	P10senF	34.4
P. hanceana (loureiroi)	F	RIV 6822 PL	USDA, CA	N/A	40.88
P. hanceana (loureiroi)	M	RIV 6789 PL	USDA, CA	N/A	33.8
P. hanceana (loureiroi)	F	RIV 6790 PL	USDA, CA	N/A	32.77
P. hanceana (loureiroi)	M	RIV 6793 PL	USDA, CA	N/A	30.81

P. paludosa	M	RIV 6818 PL	USDA, CA	N/A	31.35
P. reclinata	F	RIV 6812 PL	USDA, CA	N/A	30.73
P. sylvestris	F	RIV 2256 PL	USDA, CA	N/A	43.32
P. sylvestris	M	RIV 2249 PL	USDA, CA	N/A	43.09

* *P.* denotes *Phoenix*

Supplementary Table 2. **A table of contigs/scaffolds used in this study**

Name	X, Y, or Autosomal	Length (bp)	NCBI Accession
dpS5Y	Y	567483	MH680965
dpS5X	X	566497	MH680976
dpS5560CH1	Autosomal	1371428	MH680988
dpS5560CH2	Autosomal	1366600	MH680991
dpS5784H1	X	706543	MH680973
dpS5784H2	Y	705158	MH680984
dpS12Y	Y	89036	MH680966
dpS12X	X	89768	MH680977
dpS5828CH1	Autosomal	225201	MH680987
dpS5828CH2	Autosomal	225793	MH680990
dpS5887CH1	Autosomal	30183	MH680989
dpS5887CH2	Autosomal	30198	MH680992
dpS10Y	Y	34743	MH680969
dpS10X	X	34736	MH680980
dpS9X	X	43944	MH680975
dpS9Y	Y	41807	MH680986
dpS6X	X	89164	MH680970
dpS6Y	Y	91697	MH680981
dpS8Y	Y	105672	MH680967
dpS8X	X	104842	MH680978
dpS6284H1	Y	144017	MH680968
dpS6284H2	X	143984	MH680979
dpS11X	X	27585	MH680974
dpS11Y	Y	27658	MH680985
dpS6694H1	Y	147216	MH680971
dpS6694H2	X	147682	MH680982
dpS4X	X	201024	MH680972
dpS4Y	Y	201034	MH680983
dpB13X	X	198875	MH680995
dpB3X	X	282833	MH680993
dpB1X	X	137710	MH680996
dpBLOGautosomal1	Autosomal	101049	MH680997
dpBLOGautosomal2	Autosomal	135777	MH680964
dpB7X	X	349331	MH680994
dpB3Y	Y	289622	MH681003
dpBCYPlike	Y	274394	MH680999
dpB1Y	Y	130775	MH681004
dpBGPATlike	Y	238144	MH681000
dpBLOGlike	Y	111099	MH681001
dpB2Y	Y	781564	MH681002
dpS2X	X	303122	MH680998

Supplementary Table 3. PCR primers used for identifying male specific regions

Primer name	Primer sequence	Gene/Contig amplified	Amplicon size(bp)	Genic amplicon
dpBCYP1F	5'- AGTGAGTTTCAACTACTACGAT -3'	CYP contig	372	No
dpBCYP1R	5'- GTCATAAACTTGACTAAAGACC -3'	Cyp contig		
dpBCYP2F	5'- CTTCGATTTTCTCTTTGATCTC -3'	CYP contig	418	No
dpBCYP2R	5'- TCAATCCCTTAAACCACTAAAC -3'	Cyp contig		
dpBGPAT1F	5'- AGAAAACCTGATATGCTCTCTG -3'	GPAT contig	450	Yes
dBpGPAT1R	5'- TGTGATGCACTTGGTAACTACT -3'	GPAT contig		
dpBGPAT2F	5'- GGGTCCATTATAAAGTCTTGAA -3'	GPAT contig	550	No
dBpGPAT2R	5'- GCGTGTGATATACATTGTCCTA -3'	GPAT contig		
dpBLOG1F	5'- ATGTAGCTAGACAACCACAACA -3'	LOG contig	350	No
dpBLOG1R	5'- CAGCTTATCAGATGACACAATC -3'	LOG contig		
dpBCLOGauto_1F	5'- TGCTTATGTTCTCTTGTCTACG -3'	Autosomal log	440	No
dpBCLOGauto_1R	5'- ATCATGGGTGAGAGAGAGTTAC -3'	Autosomal log		
dpBCLOGauto_2F	5'- ATGATGAGAGAGCTATTGTTCA -3'	Autosomal log	365	Yes
dpBCLOGauto21R	5'- AATCATCTGATCAAAATGAGAG -3'	Autosomal log		
CYP703_ORF_1F	5'- GAATGGTAAGATCTTGAAGTCC -3'	Cyp Gene	1500	Yes
CYP703_ORF_1R	5'- TAGATGTGGTTCTTGTGTCTGT -3'	Cyp Gene		

Supplementary References

1. Beisson, F., Li, Y., Bonaventure, G., Pollard, M. & Ohlrogge, J. B. The Acyltransferase GPAT5 Is Required for the Synthesis of Suberin in Seed Coat and Root of Arabidopsis. *PLANT CELL ONLINE* **19,** 351–368 (2007).
2. Men, X. *et al.* Glycerol-3-Phosphate Acyltransferase 3 (OsGPAT3) is required for anther development and male fertility in rice. *J. Exp. Bot.* **68,** 513–526 (2017).
