# Supplementary material for: Genus-wide sequencing supports a two-locus model for sex-determination in Phoenix
Source: Nat Commun. 2018 Sep 28;9:3969. doi: 10.1038/s41467-018-06375-y (PMC6162277; doi:10.1038/s41467-018-06375-y)
Supplement: Supplementary file 3 — Descriptions of Additional Supplementary Files [file 41467_2018_6375_MOESM3_ESM.pdf]

Descriptions of Additional Supplementary Files

File Name: Supplementary Dataset 1
Description: A text file containing 16bp kmers identified in all 13 species male but absent in 14 Phoenix females.
